# Linkage disequilibrium compared between five populations of domestic sheep

**DOI:** 10.1186/1471-2156-9-61

**Published:** 2008-09-30

**Authors:** Jennifer RS Meadows, Eva KF Chan, James W Kijas

**Affiliations:** 1 CSIRO Livestock Industries, Level 5 Queensland Bioscience Precinct, 306 Carmody Road, St Lucia 4067, Australia; 2 University of New England, School of Rural Science and Agriculture, Armidale 2351, Australia

## Abstract

**Background:**

The success of genome-wide scans depends on the strength and magnitude of linkage disequilibrium (LD) present within the populations under investigation. High density SNP arrays are currently in development for the sheep genome, however little is known about the behaviour of LD in this livestock species. This study examined the behaviour of LD within five sheep populations using two LD metrics, D' and x^2'^. Four economically important Australian sheep flocks, three pure breeds (White Faced Suffolk, Poll Dorset, Merino) and a crossbred population (Merino × Border Leicester), along with an inbred Australian Merino museum flock were analysed.

**Results:**

Short range LD (0 – 5 cM) was observed in all five populations, however the persistence with increasing distance and magnitude of LD varied considerably between populations. Average LD (x^2'^) for markers spaced up to 20 cM exceeded the non-syntenic average within the White Faced Suffolk, Poll Dorset and Macarthur Merino. LD decayed faster within the Merino and Merino × Border Leicester, with LD below or consistent with observed background levels. Using marker-marker LD as a guide to the behaviour of marker-QTL LD, estimates of minimum marker spacing were made. For a 95% probability of detecting QTL, a microsatellite marker would be required every 0.1 – 2.5 centimorgans, depending on the population used.

**Conclusion:**

Sheep populations were selected which were inbred (Macarthur Merino), highly heterogeneous (Merino) or intermediate between these two extremes. This facilitated analysis and comparison of LD (x^2'^) between populations. The strength and magnitude of LD was found to differ markedly between breeds and aligned closely with both observed levels of genetic diversity and expectations based on breed history. This confirmed that breed specific information is likely to be important for genome wide selection and during the design of successful genome scans where tens of thousands of markers will be required.

## Background

Mapping genes of interest within animal genomes has been a lengthy and expensive task. In the past, the technique of choice has been within family linkage analysis, requiring the construction of large multigenerational pedigrees. A faster and more economical way to narrow the genetic interval surrounding a gene of interest is through whole genome scans and linkage disequilibrium (LD) mapping. The power of LD mapping lies in its ability to exploit historical recombination within populations of unrelated animals to track the sequence variations which contribute to phenotypic variation. Linkage disequilibrium refers to the ability of an allele from one marker to predict the allelic status at a second marker. The extent of LD serves to inform the number of markers required for a whole genome scan. A population with extensive LD will require a lower marker density as large tracts of the genome will be redundant to those surrounding it. Conversely if LD persists over short distances many more markers will be required to obtain the same power to detect association. Recombination events, population dynamics including drift and admixture as well as breed selection bottlenecks all serve to influence the extent of LD. With this in mind, it is important to quantify the extent of LD within different breeds as this is likely to have an impact on the success of gene mapping experiments.

The potential application of LD has prompted investigation into its magnitude and persistence within a number of livestock species including cattle [1-4], pig [5,6] and sheep [7]. A common finding is significant LD extending across tens of centimorgans. The majority of these studies have examined only one or two breeds, however recent studies in cattle have compared LD between multiple breeds [8,9]. In addition, an investigation comparing five divergent canine breeds which revealed marked differences between populations and a wide range in breed specific LD decay [10]. Sheep breeds represent a broad spectrum of both population history and phenotypic attributes. The process of sheep domestication began approximately 9000 years ago [11] and subsequent selection has occurred for such diverse traits as environmental tolerance, wool characteristics, milk yield and meat production. The result is formation of more than 1400 breeds [12]. The focus of this study was to sample multiple populations of sheep reflecting different population histories and to use microsatellites to measure the magnitude and significance of linkage disequilibrium across one ovine chromosome (OAR 18). By extrapolating the LD measured across a single chromosome to that present in the whole genome, the study aimed to provide a guide to minimum marker spacing for whole genome scans and to examine the impact of breed selection on such undertakings.

## Results

### Genetic Diversity and Population Structure

A total of 555 animals from five ovine populations were genotyped at 28 microsatellite loci. The mean amount of missing data per locus across all populations was 3.8% (WFS 2.7%; PD 3.1%; MER 3.2%; MxB 6.8%; EMAI 2.1%). Information describing the chromosomal location and the polymorphism observed at each marker is contained within Additional file 1. Analysis of genetic diversity within the five populations (Table 1) showed the Merino (MER) contained the highest genetic diversity as measured by average number of alleles observed per locus (*A*_N _= 8.13), gene diversity (*H*_E _= 0.70) and allelic richness (*A*_R _= 8.13). The MER also appeared the most distinct as measured by private allelic richness (*pA*_R _= 0.58). The closed population of Macarthur Merinos (EMAI) contained the lowest amount of diversity, with estimates of *A*_N _(3.03) *A*_R _(3.13) and *pA*_R _(0.09) less than half that of the next lowest population (Table 1). Comparison with previous estimates of sheep gene diversity [13] reveal that the commercial Merino used in this study was amongst the most diverse and the Macarthur Merino were approximately equivalent to the least diverse of ovine populations.

**Table 1:** Genetic Diversity Within Five Sheep Populations

		**Within Population Diversity**				**Population Fst**			
			
Population	n	*H*_E_	*A*_N_	*A*_R_	*pA*_R_	PD	MER	MxB	EMAI
WFS	84	0.68	6.90	7.21	0.26	0.035	0.051	0.063	0.257
PD	122	0.65	7.03	6.95	0.18		0.072	0.085	0.259
MER	126	0.70	8.13	8.13	0.58			0.043	0.183
MxB	128	0.68	7.80	7.79	0.36				0.217
EMAI	95	0.40	3.03	3.13	0.09				

Microsatellite genotypes from 28 microsatellite markers were used to estimate the following measures of genetic diversity; *H*_E _is the expected heterozygosity or gene diversity; *A*_N _is the average number of observed alleles per locus; *A*_R _is allelic richness, a measure of diversity following rarefaction for sample size; *pA*_R _is private allele richness, a simple measure of population distinctiveness. All measures were calculated using HP-RARE ver1.0 [29]. *n *is the number of individuals tested. Pair-wise estimates of *F*_ST _were calculated using the program FSTAT 2.9.3.2 .

**Figure 1:**
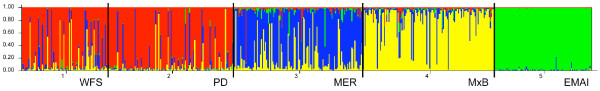
**Cluster analysis of five sheep populations**. Analysis of White Faced Suffolk (WFS), Poll Dorset (PD), Merino (MER), Merino × Border Leicester (MxB) and the Macarther Merino using STRUCTURE v2.2 [14] reveals the total genetic variation was explained with four sub-populations.

The level of relatedness between ovine populations was investigated by calculation of pair-wise *F*_ST _(Table 1). The smallest value was observed between the White Faced Suffolk (WFS) and Poll Dorset (PD) (*F*_ST _= 0.035), indicating of the five groups analysed, these two are the most closely related. The next lowest *F*_ST _was observed between the MER and MxB (*F*_ST _= 0.043). This is likely a reflection of the common Merino contribution to both populations. The highest *F*_ST _values were observed for every pair-wise combination of populations which included the EMAI animals. A cluster based method was used to estimate the minimum number of sub-populations (*K*) required to explain the total sum of genetic variation observed [14]. Figure 1 illustrates four sub-populations (*K *= 4) differentiated the MER, MxB and EMAI as distinct populations, however the fourth cluster contains both the WFS and PD. The undifferentiated genetic unit containing both the WFS and PD is in keeping with the low *F*_ST _reported for these breeds and is also consistent with breed history, as the White Faced Suffolk was founded in part by the Poll Dorset. Cluster analysis also illustrated subpopulation diversity. Figure 1 shows the EMAI group as a solid green block which is almost completely free from contribution of other sub-populations whilst MER appears to be a more heterogeneous subpopulation.

### Linkage Disequilibrium Analysis Using x^2'^

**Figure 2:**
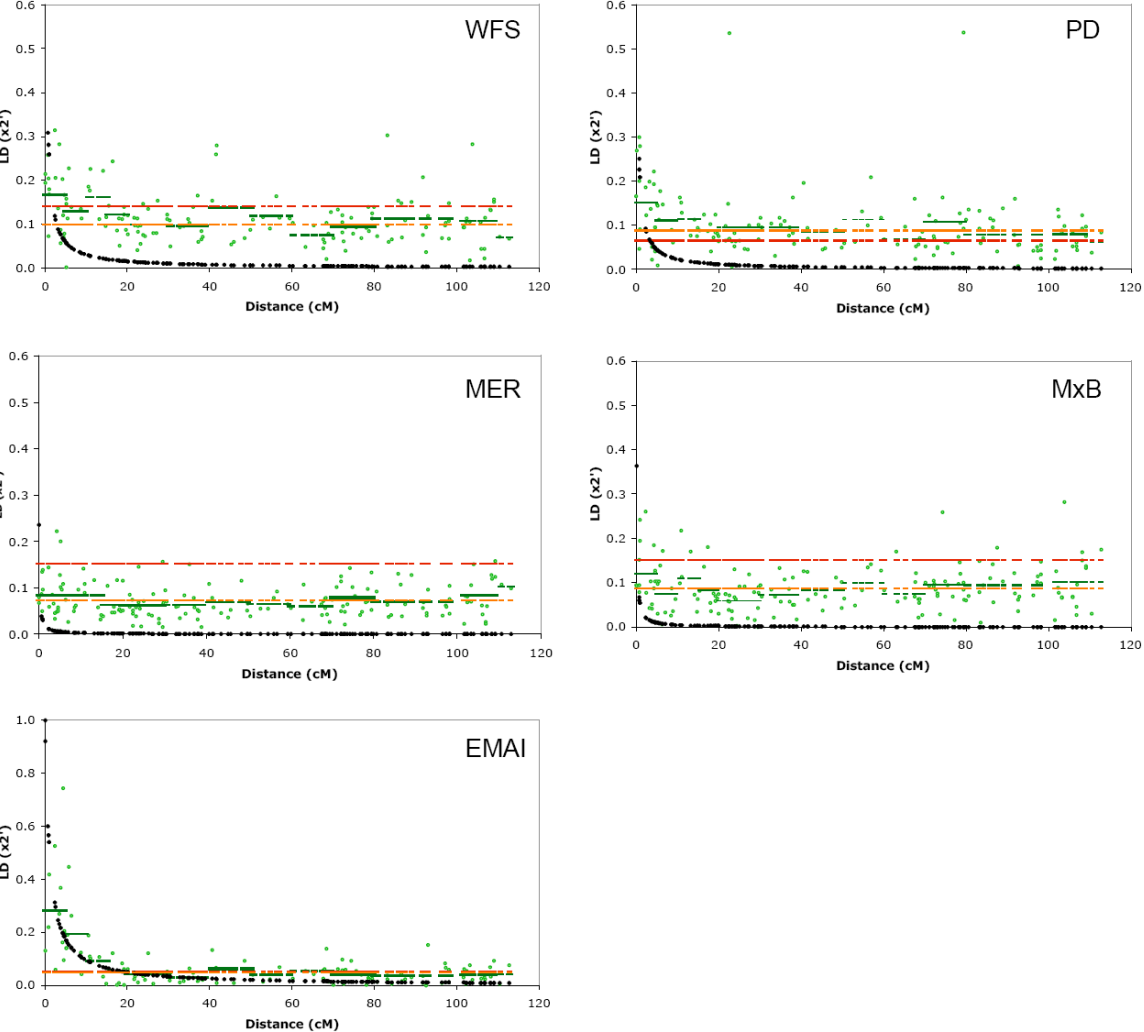
**Linkage disequilibrium (x^2'^) as a function of genetic distance**. Each population is plotted in a separate panel. The absolute values of x^2' ^(green circles) are plotted as a function of the genetic distance separating each marker pair (cM). Note the Y axis scale (x^2'^) is not the same for each population. The mean value of x^2' ^within defined distance bins is shown as horizontal green bars and contained within Table 2. The decay of LD modelled as a function of distance according to formula 3 is shown using black diamonds. Two significance thresholds are indicated using horizontal lines. The first represents the average x^2' ^value obtained between non-syntenic marker pairs (orange line) while the second represents the 5% significance threshold (red line).

Linkage disequilibrium was estimated for all marker pairs using the metric x^2'^, a standardised chi-square statistic suitable for use with multi-allelic markers [15]. The values of x^2' ^derived from chromosome 18 marker pairs were plotted as a function of increasing genetic distance (Figure 2). Figure 2 shows x^2' ^derived from syntenic marker pairs (green circles) exceeded the average derived from non-syntenic markers (orange line) for closely spaced markers in each of the five populations tested. For example, average LD for markers separated by less than 5 cM in WFS (x^2' ^= 0.167 ± 0.076) was well above the average observed using non-syntenic markers in the same population (x^2' ^= 0.099 ± 0.047; Figure 2, Table 2). Short range LD was observed in all five populations, however LD was observed to persist over larger chromosomal distances in some populations. Average LD for markers spaced up to 20 cM exceeded the non-syntenic average within the WFS, PD and EMAI populations (Table 2, Figure 2). When x^2' ^was compared against the 5% threshold for significant LD (red line, Figure 2), many fewer marker pairs display both the magnitude and significance which exceeds the critical level. This was particularly evident in MER and MxB where less than 9% of marker pair combinations had x^2' ^which exceeded the 5% threshold. The threshold limits applied here (0.05 – 0.15, Table 2) did not appear unrealistically high when compared to those applied in commercial chicken (x^2' ^range 0.07 – 0.25) [16].

**Table 2:** Mean x^2' ^with Increasing Genetic Distance

	**Population**				
	
Distance bin	WFS	PD	MER	MxB	EMAI
0–5 cM	0.167 (0.076)	0.151 (0.086)	0.084 (0.048)	0.120 (0.064)	0.283 (0.199)
5–10 cM	0.129 (0.063)	0.111 (0.056)	0.084 (0.051)	0.075 (0.051)	0.192 (0.131)
					
0–10 cM	0.156 (0.073)	0.139 (0.079)	0.084 (0.048)	0.102 (0.062)	0.250 (0.179)
10–20 cM	0.139 (0.056)	0.100 (0.032)	0.072 (0.035)	0.096 (0.054)	0.067 (0.055)
20–30 cM	0.098 (0.030)	0.096 (0.110)	0.062 (0.037)	0.060 (0.034)	0.042 (0.030)
30–40 cM	0.095 (0.033)	0.096 (0.033)	0.063 (0.033)	0.072 (0.033)	0.028 (0.017)
40–115 cM	0.105 (0.055)	0.096 (0.065)	0.073 (0.032)	0.093 (0.047)	0.042 (0.034)
					
Non-syntenic	0.099 (0.047)	0.088 (0.047)	0.073 (0.033)	0.087 (0.047)	0.048 (0.071)

*n *Marker Pairs					
Syntenic	153	153	171	171	120
Non-Syntenic	198	198	207	207	180

Critical Threshold 5%	0.141	0.065	0.151	0.151	0.053

*b*_*j *_from formula 3	0.802	1.066	9.015	4.875	0.239

Mean values for x^2' ^(standard deviation) were calculated following classification of marker pairs into distance bins. The number of both syntenic and non-syntenic marker pairs used for the calculation of mean x^2' ^are given for each population. The x^2' ^value which corresponds to the 5% level of significance is given for each population. This appears as a horizontal red line in Figure 2. The decay of LD with distance is quantified using *b*_*j *_(formula 3).

The proportion of microsatellite pairs in significant LD was determined as a function of genetic distance (Table 3). As expected, the proportion of significant LD decreased with increasing genetic distance. At short distances (< 5 cM), a high proportion of marker pairs displayed significant LD (Table 3). The highest proportion was observed within Macarthur Merinos (1.00) and lowest with the commercial Merino (0.56). In each population, with the exception of MER, the proportion of marker pairs in significant LD exceeded the non-syntenic fraction for pairs up to 20 cM apart.

**Table 3:** The Proportion of Marker Pairs in Significant LD

	**Population**				
	
**Distance bin**	**WFS**	**PD**	**MER**	**MxB**	**EMAI**
0–5 cM	13/18 (0.72)	15/18 (0.83)	10/18 (0.56)	11/18 (0.61)	12/12 (1.00)
5–10 cM	6/8 (0.75)	6/8 (0.75)	4/12 (0.33)	3/12 (0.25)	6/7 (0.86)
					
0–10 cM	19/26 (0.73)	21/26 (0.81)	14/30 (0.47)	14/30 (0.47)	18/19 (0.95)
10–20 cM	10/16 (0.63)	8/16 (0.50)	2/16 (0.13)	4/16 (0.25)	8/14 (0.57)
20–30 cM	6/19 (0.32)	12/19 (0.63)	7/24 (0.29)	1/24 (0.04)	3/12 (0.25)
30–40 cM	2/12 (0.17)	6/12 (0.50)	2/14 (0.14)	1/14 (0.07)	1/11 (0.09)
40–115 cM	9/80 (0.11)	33/80 (0.41)	3/87 (0.03)	10/87 (0.11)	8/64 (013)
					
Non-syntenic	24/198 (0.12)	83/198 (0.42)	28/207 (0.14)	19/207 (0.10)	22/180 (0.12)

The number of marker pairs with significant x^2' ^(p < 0.05) is given before the total number of markers tested for each bin and population. The proportion is given in brackets.

### Rate of LD Decay Compared Between Breeds

To examine the decline in LD, decay with distance was modelled and plotted (black line, Figure 2) and the coefficient of decay (*b*_*j*_) used to quantify this curve for each population. The value for *b*_*j *_is inversely proportional to the extent of LD, meaning high values of *b*_*j *_indicate a low persistence of disequilibrium with distance [16]. Table 2 shows the maximum decay coefficient was observed within the MER (*b *= 9.015) followed by the MxB (4.875), PD (1.066), WFS (0.802) and EMAI (0.239).

### Linkage Disequilibrium Analysis Using D'

Linkage disequilibrium was estimated for all marker pairs using D' (see Additional files 2, 3, 4). This facilitated comparison with x^2' ^(this study) and the only other investigation of LD in sheep which employed D' [7]. The magnitude of D', plotted as a function of genetic distance, revealed the expected decline with increasing distance was only clearly evident in the Macarthur Merino and Poll Dorset populations (Additional file 2). Examples of strong LD (D' > 0.5) can be seen at long range (> 30 cM) in several populations, consistent with previous studies in sheep and other livestock species [1,4,7]. Comparison of D' against the 5% critical threshold for significance revealed low levels of average LD in the MER and MxB, even over short genetic distances (red line, Additional file 2). Estimation of D' between non-syntenic marker pairs revealed high levels in all five populations, ranging from 0.266 ± 0.07 in the Poll Dorset to 0.322 ± 0.11 in the Merino × Border Leicester (Additional file 3). The coefficient of LD decay (*b*_*j*_) was calculated for each population. Despite the marked variation in population heterozygosity and genetic variability shown in the diversity and structure analyses (Figure 1, Table 1), *b*_*j *_was approximately the same in all five groups of animals (0.027 – 0.031; Additional file 3).

### Predictions for Genome Wide Association Studies

The chance of detecting LD between a marker and QTL was estimated given the observed levels of marker-marker LD (x^2'^) using a probabilistic relationship (see equation 4). This used the proportion of marker pairs which display LD in a given range (LD_*R*_) to estimate the probability of detecting marker-QTL LD (P_*R*_). A genome scan performed using unrelated animals and markers spaced at 2 cM intervals is predicted to identify 99% of QTL within the Macarthur Merino population (LD_*R *_= 0.58; *mR *= 5; T set to x^2' ^> 0.2; calculation 1, Table 4). The probability of detecting the same QTL within commercial Merinos was dramatically lower at 25% (LD_*R *_= 0.06, Table 4). For WFS and PD, the probability remained high at 91% and 80% respectively (LD_*R *_= 0.39 and LD_*R *_= 0.58, Table 4). The same equation was used to estimate the number of markers required to achieve a 95% probability of detecting LD between a marker and QTL (P_*R *_= 0.95; calculation 2, Table 4). For the population which displayed the highest rate of LD decay (*b *= 9.015), a total of 35,000 markers would be required at 0.1 cM intervals across the genome. This minimum marker number is reduced 5 – 8 fold when the other commercial sheep populations are considered (Table 4). The predictions for genome wide association studies were revisited with population specific LD thresholds taken from the 5% critical value. This served to lower the LD threshold in all populations and as a result the probability of finding QTL and the minimum marker spacing distance increased in most populations (Table 4). The trends observed between populations remained the same.

**Table 4:** Predictions for Genome Wide Associations

	**Calculation 1**			**Calculation 2**		
		
**Population**	***T***	**LD_*R*_**	**P_*R*_**	***mR***	**M**	**Total M**
WFS	0.2	0.39	0.91	6.08	0.82	4,268
PD	0.2	0.28	0.80	9.20	0.54	6,481
MER	0.2	0.06	0.25	52.4	0.10	35,000
MxB	0.2	0.11	0.45	25.4	0.20	7,000
EMAI	0.2	0.58	0.99	3.42	1.46	2,397
						
WFS	0.141	0.61	0.99	3	1.58	2,215
PD	0.065	0.78	1.00	2	2.51	1,394
MER	0.151	0.06	0.25	52	0.10	35,000
MxB	0.151	0.28	0.80	9	0.54	6,481
EMAI	0.053	1.00	1.00	na	na	na

In calculation 1, the threshold (T) was set to 0.2 or the empirically derived 5% significance threshold for each population. This allowed the value for LD_*R *_to be taken from the dataset and used to calculate P_*R*_. The range (R) was set to 0 – 5 cM in each case and *mR *= 5. In calculation 2, P_*R *_was set to 0.95 in each case and the thresholds used were the same as for calculation 1 which resulted in use of the same values for LD_*R*_. This allowed the number of markers (*mR*) for size range (R = 5) to be calculated. *mR *was converted into the required marker spacing in cM (M) and the total number of markers required for a genome scan (Total M) for each population. The calculation was not applicable (na) where LD was equal to 1.

## Discussion

The magnitude of linkage disequilibrium (LD) and its decay with distance was measured within five sheep populations across a single chromosome (OAR18). Studies which use multi-allelic markers to measure LD in livestock species have mainly calculated D' [1,2,5], however more recent investigations have promoted use of the metric x^2' ^[15,16]. Comparison between metrics in this study revealed the average magnitude of D' was higher than x^2' ^for a given genetic distance (Table 2 and Additional file 3) and many more marker pairs had elevated values (LD > 0.60) using D'. This variance between measures has been reported previously and likely reflects the theoretical expectation that rare alleles and unobserved haplotypes tend to inflate D' but not x^2' ^[16-18]. The inflation of D' values also appeared between non-syntenic (NS) marker pairs. For the five sheep populations tested, 0 – 14% of NS pairs had D' > 0.5. When NS LD was calculated using x^2' ^however, 0 – 1.6% of marker pairs reported x^2' ^> 0.5, a nine fold reduction in apparent NS LD. This difference is smaller than the dramatic reduction reported by [16], where a 100 fold decrease in NS LD was observed within commercial chicken populations. The nine fold reduction observed in this study is still important, as artificially high levels of background LD are expected to result in a proportionate increase in the rate of false positive associations reported for whole genome scans. The conclusion is therefore that D' is to be avoided as it tends to reduce the power to identify true association where marker spacing is either dense (fine mapping) or sparse (current microsatellite based genome scans).

Only one previous investigation reported on the level of LD found within sheep populations [7]. These authors described high LD extending over tens of centimorgans and highlighted the sensitivity of D' to both rare alleles and marker heterozygosity. Comparison with this study necessitated the use of D', and direct comparison between the studies should be treated with caution due to differences in sample size, breed, population structure and the molecular markers used. A common finding to both investigations was of significant LD extending across large genetic distances. The proportion of marker pairs in significant LD persisted well above the NS-LD rate for distances up to 20 cM or more within some, but not all, of the populations tested here (Additional file 4). This lends support to the original finding of [7] by showing some sheep populations contain extensive LD.

The behaviour of LD, measured with the x^2' ^metric, was found to differ markedly between breeds. Table 2 quantifies this difference by reporting a wide range of solutions to *b*_*j*_, the coefficient of LD decay, for the five populations. LD decayed fastest within the commercial Merino (*b *= 9.02). Conversely, LD persisted over the largest distance and decayed slowest within the Macarthur Merino population (*b *= 0.24). This neatly fits both the known breed history for each population and the objective measures of genetic diversity (Table 1). For example, the Merino is an old breed, the foundation of which in Australia is known to contain contributions from numerous European, Asian and African breeds [19,20]. The levels of allelic richness and gene diversity observed place the breed amongst the most diverse sheep populations tested to date (Table 1) [13]. The finding that this high level of diversity coincided with the sharpest decline in LD suggests historic recombination and a large effective population size are likely to be responsible. At the other extreme, the Macarthur Merinos have been maintained as a closed museum flock. The animals are descendants of a small number of rams imported into the Australian colonies by John Macarthur in the early 19^th ^century [21]. The very low estimates of genetic diversity observed support anecdotal information indicating that little or no introgression into the flock has occurred. The persistence of LD over large distances was therefore not surprising and suggests a small effective population may have acted to preserve LD. The White Faced Suffolk (WFS) and Poll Dorset (PD) had intermediate coefficients of decay (WFS *b *= 0.802; PD *b *= 1.066; Table 2). In the past 100 years, both populations have undergone bottlenecks during breed formation. The WFS was developed during the 1970s in an attempt to remove the black pigmentation from the head and legs of the Suffolk [22]. Similarly, the PD was developed from the Dorset beginning in the 1930s with the aim to select against horns. In each case, breed foundation necessarily reduced the effective population size [22]. The result is a reduction in the number of haplotypes observed compared with the commercial Merino and an intermediate decline in LD as a function of distance. It is also possible selection may also played a role in generating the observed differences in LD. Each of the closely spaced microsatellites reside in a genomic region known to harbour loci which influence muscularity [23,24]. This is an important consideration given some of the breeds have been selected for muscularity (WFS, PD) more intensively than others (eg MER). Taken together, the comparison between populations indicate that LD behaves in a breed specific manner and that simple indices of genetic diversity appear to serve as predictors.

The extent of LD observed within each population was used to make predictions about marker spacing and the likelihood of detecting QTL in genome wide association studies. Table 4 shows that, dependant on the population used, microsatellite markers are required at 0.1 – 2.5 centimorgans intervals to detect QTL with high confidence. This suggests LD mapping within closed populations containing low diversity, such as long term selection lines, can be successfully performed using the existing set of approximately 1500 microsatellites [25,26]. Populations in which LD decays much more sharply will require many more microsatellites than currently available, with approximately 35,000 required for LD mapping within the commercial Merino (Table 4). Given the prohibitively high cost associated with genotyping such a large number of microsatellites, future genome wide association experiments will utilize SNP markers. It was not possible to draw any conclusion regarding the number of SNP which will be required, due to differences in information content, mutation rate and genomic distribution when SNP are compared with microsatellites. The microsatellite based projections should be considered with caution as they rely on certain assumptions. Foremost amongst these is that the magnitude and significance of LD observed across chromosome 18 is representative of the entire ovine genome. Several studies have demonstrated considerable variation in LD between chromosomes in human [27], cattle [18], deer [17] and pig [5]. The projections were also reliant on a low level of statistical significance and the requirement for only modest levels of LD between markers. Association studies which used these thresholds would likely have a high rate of false positive findings and fail to detect QTL with small effects. In addition, the extent of LD may vary significantly along the length of individual chromosomes, creating LD 'holes' which display very low levels of LD in the presence of tightly spaced markers [27]. Finally, marker – marker LD has been considered the equivalent of marker – QTL LD. Comparison between metrics revealed x^2' ^best reflects marker – QTL LD [15] however the current analysis does not consider sample size or the size of QTL effects. The frequency and severity of these phenomena are yet to be described within the ovine genome, meaning this study is likely to be calibrated by subsequent experimentation using high density genome wide SNP panels.

## Conclusion

Knowledge concerning the behaviour of LD is important for performing genome wide association analysis and the emerging objective of genomic selection. Genomic selection involves the prediction of molecular estimated breeding values (mEBV) based on markers spread across the genome [28]. The major finding of this study is that the magnitude and significance of LD varies markedly between sheep populations. This makes information concerning LD between breeds important. For example, a molecular EBV generated within one breed (eg Poll Dorset) may have limited use in a second breed where the structure of LD is different (eg Merino). Conversely, Poll Dorset derived mEBVs are likely to have higher accuracy within closely related breeds which share a similar LD structure (eg White Faced Suffolk). The characterisation of LD across OAR 18 within these historically and genetically different sheep breeds also has implications for association mapping, confirming that tens of thousands of markers will be required for genome scans.

## Methods

### Animal Resources

The study consisted of 460 Australian commercial sheep from four populations; White Faced Suffolk (WFS; n = 84), Poll Dorset (PD; n = 122), Merino (MER; n = 126) and Merino × Border Leicester (MxB; n = 128). Animals were selected from between 3 and 11 properties across Australia to ensure the recruitment of as many unrelated individuals as possible. The MER is a wool breed, the PD and WFS meat breeds and the MxB a terminal composite which has been selected for both wool and meat production. A fifth population was also included in the study. The Elizabeth Macarthur Agricultural Institute Merinos (EMAI; n = 95) are maintained as descendants of the original nineteenth century Macarthur Merinos and are a single, closed flock. DNA from the WFS, PD, MER and MxB was prepared from whole blood using QIAamp DNA mini kits (QIAGEN, Australia) following the manufacture's instructions, whilst DNA from each EMAI animal was extracted using standard phenol/chloroform methods.

### Marker Selection and Genotyping

Two panels of microsatellites were used. Microsatellite panel 1 (MSP1) consisted of nineteen markers selected to span 113 cM of ovine chromosme (OAR) 18. Marker locations (in cM) were taken from the CompLDB integrated map [26]. The average distance separating marker pairs was 6.2 cM, with the smallest interval 0 cM and the largest 30.5 cM. Panel 2 (MSP2) was composed of nine microsatellites, each located on different autosomes, plus *hh47 *from MSP1. MSP2 was used to estimate levels of non-syntenic LD. The forward primer of each marker pair was fluorescently labelled and after multiplex PCR was performed, the products were separated using an ABI 3130 × l Genetic Analyser (Applied Biosystems, USA). GeneMapper v3.7 software (Applied Biosystems, USA) was used for allele sizing and binning. The name, genomic location, observed allelic size range and polymorphism associated with each marker is presented in Addition file 1.

### Genetic Analysis of Genetic Diversity

Four indices of genetic diversity were used to compare the amount of diversity within each ovine population. Calculations of gene diversity (*H*_E_), average number of alleles per locus (*A*_N_), allelic richness (*A*_R_) and private allelic richness (p*A*_R_) were performed using the complete data set (MSP1 and MSP2) in HP-RARE v1.0 [29]. FSTAT 2.9.3.2  was used to evaluate population relatedness using pair-wise estimates of *F*_ST_. The presence of population substructure was investigated using MSP2 data and an admixture ancestry model-based clustering method as implemented in STRUCTURE v2.2 [14]. Three replicates of one to five subpopulations (*K *= 1 – 5) were performed using 50,000 Markov chain steps after a burn-in period of 20,000 steps.

### Analysis of Linkage Disequilibrium

Two measures were considered. The first metric, x^2' ^(formula 1), has recently been proposed as the measure of choice for use with multi-allelic markers such as microsatellites [15]. The second metric, D' (formula 2), was first described by Hedrick [30] as a multi-allelic extension of Lewontin's D'_*ij *_[31]. D' was implemented by the only other published study to empirically measure ovine LD [7].

(1) *x*^2' ^= *x*^2^/[2N(*n *- 1)]

where,

$$x^{2}=N\sum_{i}\sum_{j}\left(D_{ij}^{2}/[(P(A_{i}))(P(B_{j}))\right]$$

and

*D*_*ij *_= *P*(*A*_*i*_*B*_*j*_) - *P*(*A*_*i*_)*P*(*B*_*j*_) where *P*(*A*_*i*_) is the frequency of allele *i *at marker A, *P*(*B*_*j*_) is the frequency of allele *j *at marker B. *N *is the population size and *n *is the number of alleles at the marker with the smaller number of alleles.

(2) $$\begin{array}{l} D'=\sum_{i}\sum_{j}P(A_{i})P(B_{j})|D_{ij}/D_{ij}^{\max}|\\ D_{ij}^{\max}=\min[P(A_{i})P(B_{j}),(1-P(A_{i}))(1-P(B_{j}))]\text{ when }D_{ij}<0,\\ D_{ij}^{\max}=\min[P(A_{i}))(1-P(B_{j})),(1-P(A_{i}))(P(B_{j}))]\text{ when }D_{ij}>0. \end{array}$$

Both x^2' ^and D' require two-marker haplotype frequency estimation. This was performed using the Expectation-Maximisation (EM) algorithm and 20 initial conditions for each of 5000 permutation tests. The maximum likelihood estimate of haplotype frequencies was then used to estimate D' and x^2'^. The EM algorithm, D' and associated p-value calculations were implemented in PyPOP release 0.6.0 [32] whilst the calculation of x^2' ^was performed with *R *statistical software [33]. LD derived from non-syntenic marker pairs was used to determine the critical levels of significance for each metric and population. This was achieved by ranking the p-values and selecting the LD value corresponding to the 5% significance threshold in each population. Theory states LD is negatively correlated with genetic distance [34]. This principle was examined graphically by plotting each metric as a function of distance (in centimorgans). The decay in LD was quantified by fitting the following formula to the observed data. [16]

(3) LD_*ij *_= 1/(1 + 4*b*_*j*_*d*_*ij*_) + *e*_*ij *_

where LD_*ij *_is the LD between microsatellite pair *i *of population *j*, separated by genetic distance (in cM) *d*_*ij*_, and where *b*_*j *_expresses LD decay with distance for population *j*, and *e*_*ij *_equates to the model residual. Parameter *b*_*j *_was calculated using the *nls *function set in *R*.

### Predictions for Genome Wide Association Analysis

Calculations regarding genome wide association studies were made using formula 4. The proportion of marker pairs within a given cM distance range (*R*) which had x^2' ^values exceeding a defined threshold (*T*) was termed LD_*R*_. The number of markers in this range was denoted *MR *and the probability of finding QTL with LD > T with at least one marker in the given range is (P_*R*_). The relationship between each is given in formula 4 as: [16]

(4) P_*R *_= 1 - (1-LD_R_)^*MR *^

Two separate questions were addressed (reported as calculation 1 and 2, Table 4). Firstly, the probability of detecting a QTL was estimated given observed levels of LD within each population. For this calculation, marker spacing was assumed to be 2 cM, as this is the approximate situation in sheep (1,500 microsatellites and genome size of 3,500 cM [26]). At 2 cM intervals, a randomly positioned QTL would be within 5 cM of approximately 5 markers (ie for distance range (*R*) 0 – 5 cM; number of markers (*MR*) = 5). The value of LD_*R *_was determined empirically where *T *was set to either x^2' ^> 0.2 or the 5% critical threshold for significance. *T *> 0.2 represents the threshold estimate of detecting QTL between SNP taken from [28]. Zhao and colleagues [15] illustrated that the metric of measuring SNP LD, r^2 ^and x^2' ^are comparable. The second question examined the number of markers (*MR*) required to obtain a 95% probability of detecting QTL given the observed magnitude of LD in each population (ie *R *= 0 – 5 cM; P_*R *_= 0.95). The number of markers was converted into the total required for a genome scan assuming a genome size of 3,500 cM.

## Authors' contributions

JRSM performed the DNA extraction and genotyping, conducted data analysis and drafted the manuscript. EKRC performed LD analysis and JWK conceived the study and prepared the manuscript.

## Supplementary Material

Additional file 1 Summary information for the 28 microsatellite markers used in the estimation of linkage disequilibrium. Click here for file

Additional file 2 Linkage disequilibrium (D') as a function of genetic distance within five sheep populations. Click here for file

Additional file 3 Mean D' as a Function of Increasing Genetic Distance. Click here for file

Additional file 4 The Proportion of Marker Pairs in Significant LD Using D'. Click here for file

## References

[B1] Farnir F, Coppieters W, Arranz JJ, Berzi P, Cambisano N, Grisart B, Karim L, Marcq F, Moreau L, Mni M, Nezer C, Simon P, Vanmanshoven P, Wagenaar D, Georges M (2000). Extensive genome-wide linkage disequilibrium in cattle. Genome Res.

[B2] Tenesa A, Knott SA, Ward D, Smith D, Williams JL, Visscher PM (2003). Estimation of linkage disequilibrium in a sample of the United Kingdom dairy cattle population using unphased genotypes. J Anim Sci.

[B3] Khatkar MS, Collins A, Cavanagh JA, Hawken RJ, Hobbs M, Zenger KR, Barris W, McClintock AE, Thomson PC, Nicholas FW, Raadsma HW (2006). A first-generation metric linkage disequilibrium map of bovine chromosome 6. Genetics.

[B4] Odani M, Narita A, Watanabe T, Yokouchi K, Sugimoto Y, Fujita T, Oguni T, Matsumoto M, Sasaki Y (2006). Genome-wide linkage disequilibrium in two Japanese beef cattle breeds. Anim Genet.

[B5] Nsengimana J, Baret P, Haley CS, Visscher PM (2004). Linkage disequilibrium in the domesticated pig. Genetics.

[B6] Harmegnies N, Farnir F, Davin F, Buys N, Georges M, Coppieters W (2006). Measuring the extent of linkage disequilibrium in commercial pig populations. Anim Genet.

[B7] McRae AF, McEwan JC, Dodds KG, Wilson T, Crawford AM, Slate J (2002). Linkage disequilibrium in domestic sheep. Genetics.

[B8] Gautier M, Faraut T, Moazami-Goudarzi K, Navratil V, Foglio M, Grohs C, Boland A, Garnier JG, Boichard D, Lathrop GM, Gut IG, Eggen A (2007). Genetic and haplotypic structure in 14 European and African cattle breeds. Genetics.

[B9] McKay SD, Schnabel RD, Murdoch BM, Matukumalli LK, Aerts J, Coppieters W, Crews D, Dias Neto E, Gill CA, Gao C, Mannen H, Stothard P, Wang Z, Van Tassell CP, Williams JL, Taylor JF, Moore SS (2007). Whole genome linkage disequilibrium maps in cattle. BMC Genet.

[B10] Sutter NB, Eberle MA, Parker HG, Pullar BJ, Kirkness EF, Kruglyak L, Ostrander EA (2004). Extensive and breed-specific linkage disequilibrium in Canis familiaris. Genome Res.

[B11] Ryder ML, Mason IL (1984). Sheep. Evolution of Domesticated Animals.

[B12] Scherf BD, editor (2000). World Watch List for domestic animal diversity.

[B13] Tapio I, Tapio M, Grislis Z, Holm LE, Jeppsson S, Kantanen J, Miceikiene I, Olsaker I, Viinalass H, Eythorsdottir E (2005). Unfolding of population structure in Baltic sheep breeds using microsatellite analysis. Heredity.

[B14] Pritchard JK, Stephens M, Donnelly P (2000). Inference of population structure using multilocus genotype data. Genetics.

[B15] Zhao H, Nettleton D, Soller M, Dekkers JC (2005). Evaluation of linkage disequilibrium measures between multi-allelic markers as predictors of linkage disequilibrium between markers and QTL. Genet Res.

[B16] Heifetz EM, Fulton JE, O'Sullivan N, Zhao H, Dekkers JC, Soller M (2005). Extent and consistency across generations of linkage disequilibrium in commercial layer chicken breeding populations. Genetics.

[B17] Slate J, Pemberton JM (2007). Admixture and patterns of linkage disequilibrium in a free-living vertebrate population. J Evol Biol.

[B18] Thévenon S, Dayo GK, Sylla S, Sidibe I, Berthier D, Legros H, Boichard D, Eggen A, Gautier M (2007). The extent of linkage disequilibrium in a large cattle population of western Africa and its consequences for association studies. Anim Genet.

[B19] Massy C (1990). The Australian Merino.

[B20] Davis GH, Galloway SM, Ross IK, Gregan SM, Ward J, Nimbkar BV, Ghalsasi PM, Nimbkar C, Gray GD, Subandriyo, Inounu I, Tiesnamurti B, Martyniuk E, Eythorsdottir E, Mulsant P, Lecerf F, Hanrahan JP, Bradford GE, Wilson T (2002). DNA tests in prolific sheep from eight countries provide new evidence on origin of the Booroola (FecB) mutation. Biol Reprod.

[B21] Parsonson I (1998). The Australian ark: a history of domesticated animals in Australia (1788 – 1988).

[B22] Australian Meat & Livestock Corporation (1989). Handbook of Australian Livestock.

[B23] Freking BA, Murphy SK, Wylie AA, Rhodes SJ, Keele JW, Leymaster KA, Jirtle RL, Smith TP (2002). Identification of the single base change causing the callipyge muscle hypertrophy phenotype, the only known example of polar overdominance in mammals. Genome Res.

[B24] Walling GA, Visscher PM, Wilson AD, McTeir BL, Simm G, Bishop SC (2004). Mapping of quantitative trait loci for growth and carcass traits in commercial sheep populations. J Anim Sci.

[B25] Maddox JF, Davies KP, Crawford AM, Hulme DJ, Vaiman D, Cribiu EP, Freking BA, Beh KJ, Cockett NE, Kang N, Riffkin CD, Drinkwater R, Moore SS, Dodds KG, Lumsden JM, van Stijn TC, Phua SH, Adelson DL, Burkin HR, Broom JE, Buitkamp J, Cambridge L, Cushwa WT, Gerard E, Galloway SM, Harrison B, Hawken RJ, Hiendleder S, Henry HM, Medrano JF, Paterson KA, Schibler L, Stone RT, van Hest B (2001). An enhanced linkage map of the sheep genome comprising more than 1000 loci. Genome Res.

[B26] Liao W, Collins A, Hobbs M, Khatkar MS, Luo J, Nicholas FW (2007). A comparative location database (CompLDB): map integration within and between species. Mamm Genome.

[B27] Service S, DeYoung J, Karayiorgou M, Roos JL, Pretorious H, Bedoya G, Ospina J, Ruiz-Linares A, Macedo A, Palha JA, Heutink P, Aulchenko Y, Oostra B, van Duijn C, Jarvelin MR, Varilo T, Peddle L, Rahman P, Piras G, Monne M, Murray S, Galver L, Peltonen L, Sabatti C, Collins A, Freimer N (2006). Magnitude and distribution of linkage disequilibrium in population isolates and implications for genome-wide association studies. Nat Genet.

[B28] Meuwissen TH, Hayes BJ, Goddard ME (2001). Prediction of total genetic value using genome-wide dense marker maps. Genetics.

[B29] Kalinowski ST (2005). HP-Rare: a computer program for performing rarefaction on measures of allelic diversity. Mol Ecol Notes.

[B30] Hedrick PW (1987). Gametic disequilibrium measures: proceed with caution. Genetics.

[B31] Lewontin RC (1964). The interaction of selection and linkage. I. General considerations; heterotic models. Genetics.

[B32] Lancaster AK, Single RM, Solberg OD, Nelson MP, Thomson G (2007). PyPop update-a software pipeline for large-scale multilocus population genomics. Tissue Antigens.

[B33] R Development Team (2007). R: A language and environment for statistical computing.

[B34] Sved JA (1971). Linkage disequilibrium and homozygosity of chromosome segments in finite populations. Theor Popul Biol.

[B35] Park SDE (2001). Trypanotolerance in West African cattle and the population genetic effects of selection. Ph D thesis.

