# Supplementary material for: Linkage disequilibrium compared between five populations of domestic sheep
Source: BMC Genet. 2008 Sep 30;9:61. doi: 10.1186/1471-2156-9-61 (PMC2572059; doi:10.1186/1471-2156-9-61)
Supplement: Additional file 1 — Summary information for the 28 microsatellite markers used in the estimation of linkage disequilibrium. [file 1471-2156-9-61-S1.doc]

### Additional file 1

Summary information for the 28 microsatellite markers used in the estimation of linkage disequilibrium.

|  |  | **Genomic Location** | |  |  |  |
| --- | --- | --- | --- | --- | --- | --- |
| **Panel** | **Marker** | **Chromosome** | **cM** | **Allelic Range (bp)** | **N_A_** | **PIC** |
|  |  |  |  |  |  |  |
| MSP1 | mcm216 | OAR 18 | 5.9 | 176 – 216 | 17 | 0.86 |
|  | L14b2 | OAR 18 | 8.2 | 151 – 163 | 7 | 0.66 |
|  | bm1117a | OAR 18 | 11.7 | 84 – 112 | 13 | 0.81 |
|  | mcm131 | OAR 18 | 16.8 | 82 – 107 | 16 | 0.84 |
|  | bm3413 | OAR 18 | 22.3 | 177 – 197 | 10 | 0.61 |
|  | ilsts52 | OAR 18 | 35.5 | 156 – 186 | 16 | 0.82 |
|  | vh54 | OAR 18 | 41.9 | 94 – 114 | 11 | 0.72 |
|  | mcm148 | OAR 18 | 46.5 | 193 – 215 | 9 | 0.67 |
|  | hh47 | OAR 18 | 77.0 | 110 – 144 | 13 | 0.78 |
|  | bm846 | OAR 18 | 85.4 | 244 – 262 | 3 | 0.03 |
|  | ilsts54 | OAR 18 | 91.7 | 129 – 135 | 4 | 0.42 |
|  | mcm38 | OAR 18 | 92.4 | 148 – 170 | 11 | 0.74 |
|  | ob2 | OAR 18 | 94.9 | 222 – 242 | 10 | 0.77 |
|  | idgva30 | OAR 18 | 109.8 | 122 – 124 | 2 | 0.37 |
|  | oy3 | OAR 18 | 114.1 | 167 – 191 | 13 | 0.80 |
|  | mulge6 | OAR 18 | 114.1 | 183 – 195 | 7 | 0.66 |
|  | mulge5 | OAR 18 | 114.9 | 152 – 170 | 8 | 0.71 |
|  | bms1561 | OAR 18 | 114.9 | 133 – 145 | 6 | 0.17 |
|  | csap28e | OAR 18 | 118.7 | 97 – 112 | 7 | 0.57 |
|  |  |  |  |  |  |  |
| MSP2 | oarFCB128 | OAR 2 | 90.8 | 98 - 130 | 9 | 0.77 |
|  | oarCP34 | OAR 3 | 31.3 | 102 - 122 | 8 | 0.71 |
|  | mcm527 | OAR 5 | 127.4 | 149 - 179 | 14 | 0.79 |
|  | bm0757 | OAR 9 | 16.6 | 167 - 198 | 9 | 0.72 |
|  | maf65 | OAR 15 | 46.5 | 120 - 144 | 11 | 0.62 |
|  | oarFCB48 | OAR 17 | 43.6 | 136 – 177 | 12 | 0.76 |
|  | oarFCB304 | OAR 19 | 64.6 | 149 - 193 | 19 | 0.74 |
|  | oarVH72 | OAR 25 | 43.2 | 123 – 137 | 8 | 0.73 |
|  | bm6526 | OAR 26 | 16.4 | 141 - 173 | 13 | 0.79 |
|  |  |  |  |  |  |  |

The total number of observed alleles (N_A_) and the polymorphism information content (PIC) for the five combined populations was prepared using the Microstellites Tool Kit [35]. The composite map of Liao [26] was used to position all microsatellites.
