# Supplementary material for: Linkage disequilibrium compared between five populations of domestic sheep
Source: BMC Genet. 2008 Sep 30;9:61. doi: 10.1186/1471-2156-9-61 (PMC2572059; doi:10.1186/1471-2156-9-61)
Supplement: Additional file 2 — Linkage disequilibrium (D') as a function of genetic distance within five sheep populations. [file 1471-2156-9-61-S2.doc]

###
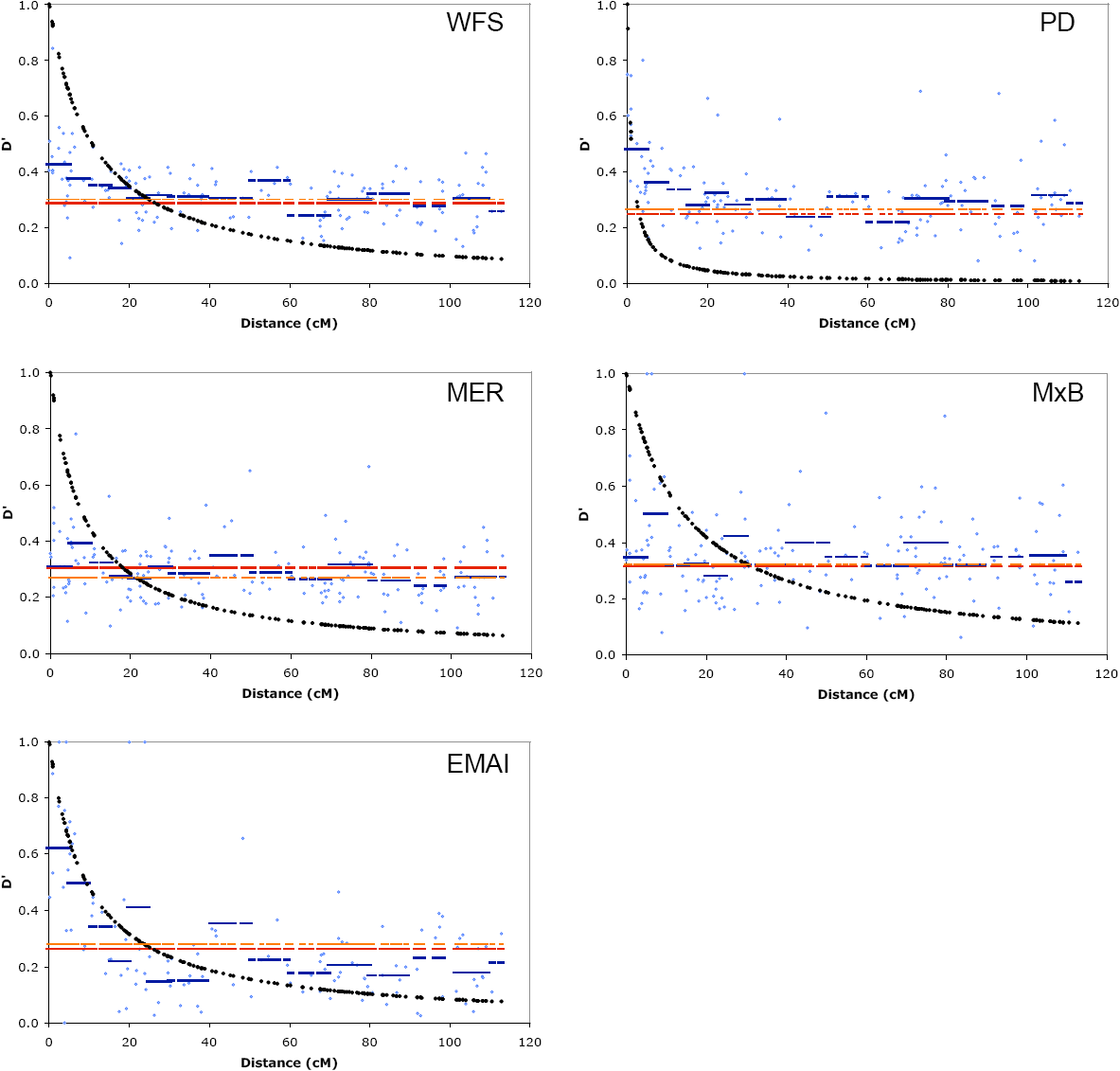


Linkage disequilibrium (D’) as a function of genetic distance within five sheep populations. The absolute value of D’ (blue diamonds) was plotted as a function of genetic distance separating each marker pair (cM). The mean value of D’ within defined distance bins is shown as horizontal blue bars. The decay of LD modelled as a function of distance according to formula 3 is shown using black diamonds. The 5% significance threshold derived from non-syntenic marker pairs is indicated using a horizontal red line and the average non-syntenic LD value is indicated with a orange line.
