# Supplementary material for: Linkage disequilibrium compared between five populations of domestic sheep
Source: BMC Genet. 2008 Sep 30;9:61. doi: 10.1186/1471-2156-9-61 (PMC2572059; doi:10.1186/1471-2156-9-61)
Supplement: Additional file 3 — Mean D' as a Function of Increasing Genetic Distance. [file 1471-2156-9-61-S3.doc]

### Additional file 3

Mean D’ as a Function of Increasing Genetic Distance

|  |  |  |  |  |  |
| --- | --- | --- | --- | --- | --- |
|  | Population | | | | |
| **Distance bin** | **WFS** | **PD** | **MER** | **MxB** | **EMAI** |
|  |  |  |  |  |  |
| 0-5cM | 0.426 (0.136) | 0.480 (0.168) | 0.310 (0.105) | 0.347 (0.146) | 0.672 (0.218) |
| 5-10cM | 0.376 (0.139) | 0.364 (0.127) | 0.393 (0.154) | 0.503 (0.160) | 0.498 (0.201) |
|  |  |  |  |  |  |
| 0-10 cM | 0.411 (0.136) | 0.444 (0.163) | 0.343 (0.131) | 0.409 (0.222) | 0.608 (0.223) |
| 10-20 cM | 0.346 (0.082) | 0.305 (0.086) | 0.297 (0.106) | 0.321 (0.093) | 0.272 (0.137) |
| 20-30 cM | 0.309 (0.072) | 0.311 (0.129) | 0.286 (0.082) | 0.340 (0.181) | 0.301 (0.333) |
| 30-40 cM | 0.312 (0.072) | 0.301 (0.105) | 0.285 (0.099) | 0.322 (0.076) | 0.151 (0.078) |
| 40-115cM | 0.299 (0.079) | 0.286 (0.112) | 0.286 (0.099) | 0.356 (0.143) | 0.209 (0.115) |
|  |  |  |  |  |  |
| Non-syntenic | 0.302 (0.072) | 0.266 (0.070) | 0.270 (0.068) | 0.322 (0.109) | 0.281 (0.216) |
|  |  |  |  |  |  |
| *n* Marker Pairs |  |  |  |  |  |
| Syntenic | 153 | 153 | 171 | 171 | 120 |
| Non-Syntenic | 198 | 198 | 207 | 207 | 180 |
|  |  |  |  |  |  |
| Critical Threshold |  |  |  |  |  |
| 5% | 0.289 | 0.250 | 0.307 | 0.316 | 0.263 |
|  |  |  |  |  |  |
| *b_j_* from formula 3 | 0.023 | 0.026 | 0.031 | 0.017 | 0.027 |
|  |  |  |  |  |  |

Mean values for D’ (standard deviation) were calculated following classification of marker pairs into distance bins. The number (*n*) of both syntenic and non-syntenic marker pairs used for the calculation of mean D’ are given for each population. The D’ value which corresponds to the 5% level of significance is given for each population. This appears as a vertical red line Additional file 2. The decay of LD with distance is quantified using *b_j_* (formula 3).
