# Supplementary material for: Linkage disequilibrium compared between five populations of domestic sheep
Source: BMC Genet. 2008 Sep 30;9:61. doi: 10.1186/1471-2156-9-61 (PMC2572059; doi:10.1186/1471-2156-9-61)
Supplement: Additional file 4 — The Proportion of Marker Pairs in Significant LD Using D'. [file 1471-2156-9-61-S4.doc]

### Additional file 4

The Proportion of Marker Pairs in Significant LD Using D’

|  |  |  |  |  |  |
| --- | --- | --- | --- | --- | --- |
|  | Population | | | | |
| Distance bin | WFS | PD | MER | MxB | EMAI |
|  |  |  |  |  |  |
| 0-5cM | 31/53 (0.58) | 36/53 (0.38) | 17/45 (0.38) | 19/53 (0.36) | 21/22 (0.95) |
| 5-10cM | 8/21 (0.38) | 10/21 (0.48) | 4/25 (0.16) | 4/26 (0.15) | 6/8 (0.75) |
|  |  |  |  |  |  |
| 0-10 cM | 39/74 (0.53) | 46/74 (0.62) | 21/70 (0.30) | 23/79 (0.29) | 27/30 (0.90) |
| 10-20 cM | 22/57 (0.39) | 17/57 (0.30) | 4/55 (0.07) | 13/60 (0.22) | 8/17 (0.47) |
| 20-30 cM | 11/34 (0.32) | 7/34 (0.21) | 8/40 (0.20) | 2/43 (0.05) | 3/13 (0.23) |
| 30-40 cM | 4/16 (0.25) | 6/16 (0.38) | 3/17 (0.18) | 1/18 (0.06) | 1/13 (0.08) |
| 40-115cM | 15/144 (0.10) | 43/144 (0.30) | 5/143 (0.03) | 18/151 (0.12) | 8/80 (0.10) |
|  |  |  |  |  |  |
| Non-syntenic | 29/270 (0.11) | 95/270 (0.35) | 32/270 (0.12) | 15/279 (0.05) | 27/198 (0.14) |
|  |  |  |  |  |  |

The number of marker pairs with significant D’ (p < 0.05) is given before the total number tested for each bin and population. The proportion is given in brackets.
